# A nationwide fluidics biobank of polytraumatized patients: implemented by the Network “Trauma Research” (NTF) as an expansion to the TraumaRegister DGU^®^ of the German Trauma Society (DGU)

**DOI:** 10.1007/s00068-019-01193-3

**Published:** 2019-07-19

**Authors:** Borna Relja, Markus Huber-Lang, Martijn van Griensven, Frank Hildebrand, Marc Maegele, Ulrike Nienaber, Daniel P. Brucker, Ramona Sturm, Ingo Marzi

**Affiliations:** 1 Department of Trauma, Hand and Reconstructive, Surgery, University Hospital Frankfurt, Goethe-University, 60590 Frankfurt, Germany; 2 Institute of Clinical- and Experimental Trauma-Immunology, University of Ulm, Ulm, Germany; 3 Experimental Trauma Surgery, Klinikum Rechts der Isar, Technical University of Munich, Munich, Germany; 4 Department of Orthopaedic Trauma, RWTH Aachen University, Aachen, Germany; 5 Department for Traumatology and Orthopedic Surgery, Cologne-Merheim Medical Centre, University of Witten/Herdecke, Cologne, Germany; 6 AUC-Academy for Trauma Surgery, Cologne, Germany; 7 University Cancer Center Frankfurt and Interdisciplinary Biomaterial Bank and Database Frankfurt, University Hospital Frankfurt, Goethe University, Frankfurt, Germany

**Keywords:** Biobank, NTF, Registry, Trauma, Research, Consortium

## Abstract

To decrypt the complexity of the posttraumatic immune responses and to potentially identify novel research pathways for exploration, large-scale multi-center projects including not only in vivo and in vitro modeling, but also temporal sample and material collection along with clinical data capture from multiply injured patients is of utmost importance. To meet this gap, a nationwide biobank for fluidic samples from polytraumatized patients was initiated in 2013 by the task force Network “Trauma Research” (Netzwerk Traumaforschung, NTF) of the German Trauma Society (Deutsche Gesellschaft für Unfallchirurgie e.V., DGU). The NTF-Biobank completes the clinical NTF-Biobank Database and complements the TR-DGU with temporal biological samples from multiply injured patients. The concept behind the idea of the NTF-Biobank was to create a robust interface for meaningful innovative basic, translational and clinical research. For the first time, an integrated platform to prospectively evaluate and monitor candidate biomarkers and/or potential therapeutic targets in biological specimens of quality-controlled and documented patients is introduced, allowing reduction in variability of measurements with high impact due to its large sample size. Thus, the project was introduced to systemically evaluate and monitor multiply injured patients for their (patho-)physiological sequalae together with their clinical treatment strategies applied for overall outcome improval.

## Background

**Fig. 1:**
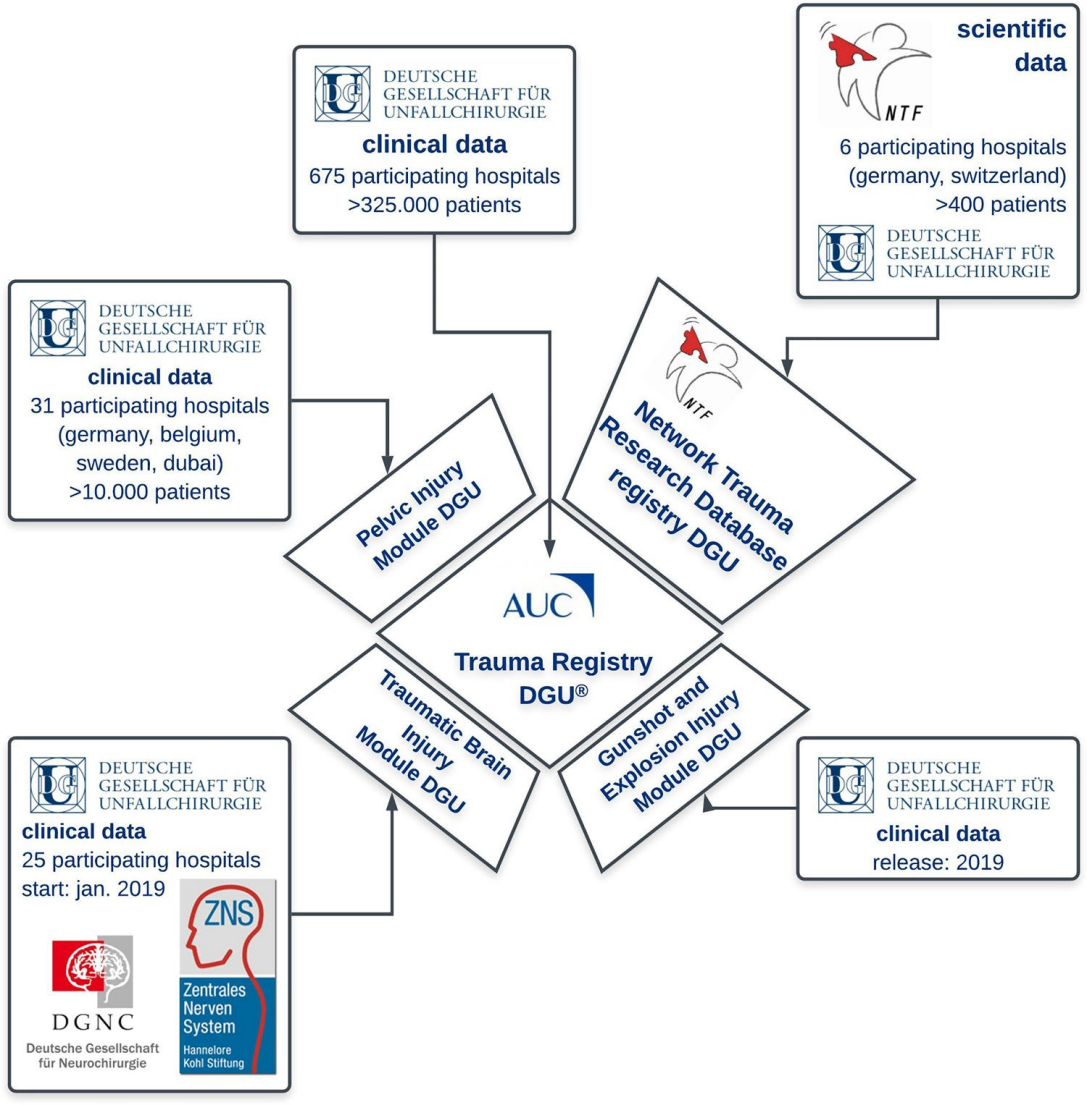
The structure of the TraumaRegister DGU^®^ of the German Trauma Society (Deutsche Gesellschaft für Unfallchirurgie, DGU) with its clinical modules, and with the unique scientific add-on “Network Trauma Research Database” provided and supervised by the task force Network “Trauma Research” (Netzwerk Traumaforschung, NTF). The AUC coordinates as a subsidiary of the DGU three different modules via the TR-DGU^®^: the Pelvic Injury Module, the Traumatic Brain Injury Module and the Gunshot and Explosion Module. TR-DGU^®^ is considered as one of the world’s largest medical trauma registries, and is the representative registry for the care of severely injured patients in Germany. More than 650 hospitals are entering data into the TR-DGU^®^ for both scientific and quality assurance purposes. Additionally, the NTF consortium conceptualized a DGU-funded add-on online Network Trauma Research (NTF) Database, which was technically translated and supported by the AUC. Adjacent to the clinical data sets, the NTF Database is including scientific data, which are obtained from the analyses of biological samples obtained from the NTF-Biobank

**Fig. 2:**
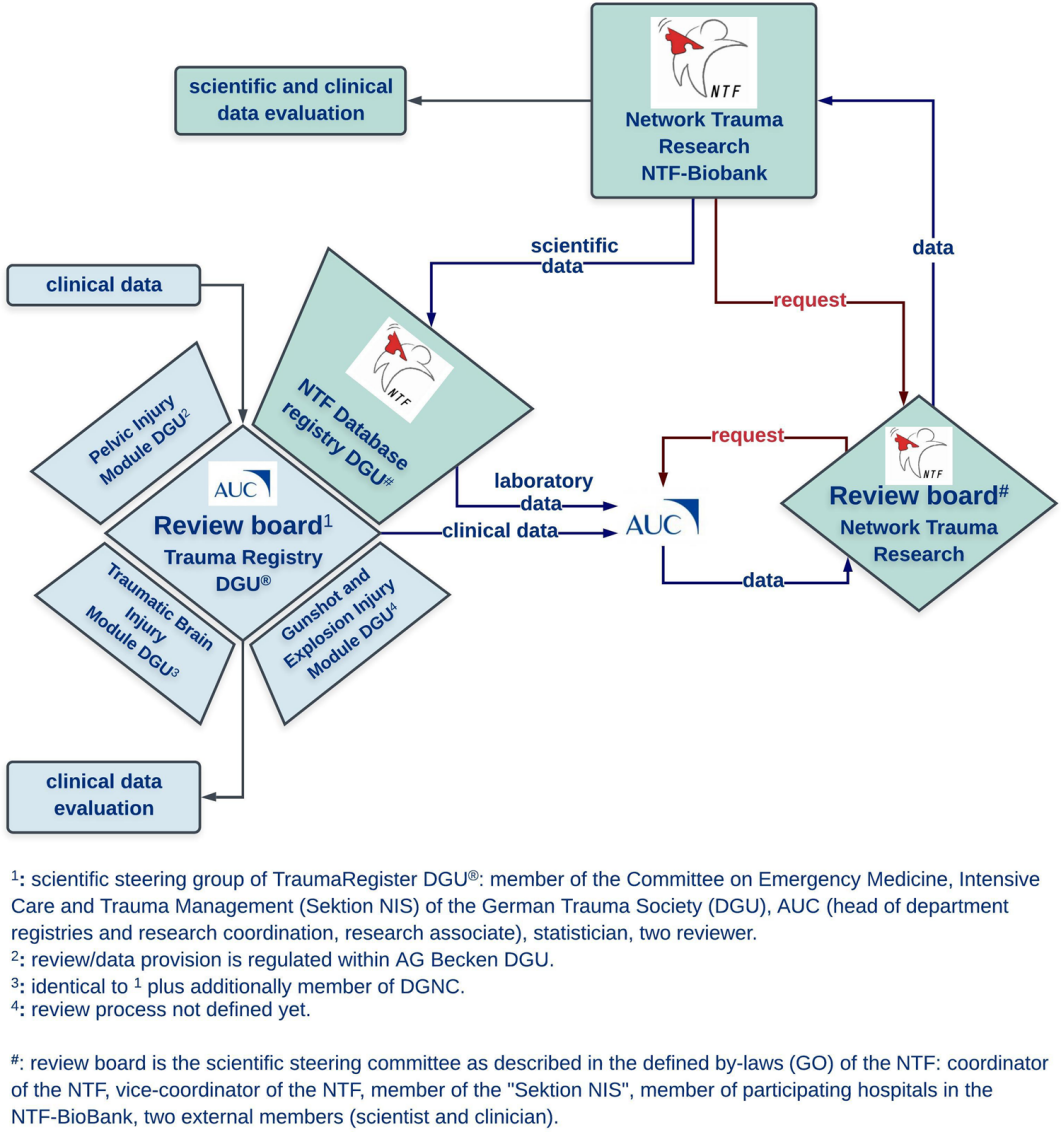
The workflow for the clinical and scientific data management with the corresponding review boards. A scientific steering group of the TR-DGU^®^ as indicated in the figure coordinates the data flow of the Trauma Registry DGU^®^. Further, each module has its own guidelines for the review and data provision as indicated. Additionally to this management of clinical data, the laboratory data from the NTF-Biobank are included in the NTF Database Registry DGU^®^. Here, the review board as well as the sample and data provision is regulated by the defined by-laws (GO) of the NTF. Thus, the storage of biological samples as well as its inventory management is secured by the NTF-Biobank and the NTF Review board. Briefly, participants in the NTF-Biobank are allowed as defined by the NTF by-laws for a project request on biosamples from the NTF-Biobank and corresponding data from the NTF Database. As indicated in the figure, upon a positive decision by the Review board of the NTF, then, the AUC will manage the provision of the corresponding laboratory and clinical data

To decrypt the complexity of the posttraumatic immune responses and to potentially identify novel research pathways for exploration, large-scale multi-center projects including not only in vivo and in vitro modeling, but also temporal sample and material collection along with clinical data capture from multiply injured patients is of utmost importance. To meet this gap, a nationwide biobank for fluidic samples from polytraumatized patients was initiated in 2013 by the task force Network “Trauma Research” (Netzwerk Traumaforschung, NTF) of the German Trauma Society (Deutsche Gesellschaft für Unfallchirurgie, DGU). The concept behind the idea of the NTF-Biobank was to create a robust interface for basic, translational and clinical research. The project was introduced to systemically evaluate and monitor multiply injured patients for their (patho-)physiological sequalae together with their clinical treatment strategies applied for overall outcome improval (Figs. 1, 2).

## Network “Trauma Research”(Netzwerk Traumaforschung, NTF)

In February 2011, a first meeting of the recently founded Network “Trauma Research” took place at the Reisensburg Castle, Günzburg [1]. Since then, numerous representatives of trauma-related research institutes and university hospitals in Germany demonstrate their main research foci in biannual national meetings to counteract the limited information about the research topics of individual research groups nationwide. The main goal of the NTF addresses joint research with the focus on traumatic injury, inflammation and coagulation on an organ and cellular level. Furthermore, the NTF provides a platform for the exchange on methodical and personal level. Also an open discussion takes place on current not only scientific problems in trauma research, i.e. the lack of junior researchers took place. Furthermore, an inclusive, intensive scientific exchange as well as the generation and workup of common hypotheses using standard operating procedures was reached. Meanwhile, the resulting clustered research activities work on clinically relevant questions in the field of trauma research. As one of the common achievements, the NTF-Biobank, which links the scientific research data directly to the matched clinical data, has been initiated in 2013.

## Current stage

Traumatic injury as a significant contributor to worldwide mortality is one of the most relevant, but neglected health concerns [2, 3]. Multiple injured patients frequently die either immediately or early after the traumatic impact within only of a few hours due to injury severity, traumatic brain injuries (TBI) and exsanguination or in the later post-injury phase due to inflammation-related complications. Such complications may affect the immune system homeostasis, and cause sepsis or (multiple) organ failure (MOF) [3–7]. While the primary impact can only be addressed by injury prevention, the later detrimental sequalae may be prevented by abrogation of the posttraumatic course [8, 9]. Over the last three decades, numerous experimental singular, double-hit and few polytrauma models combining different injury patterns have been developed and used to study and understand the basic pathophysiology of the posttraumatic sequelae in polytraumatized patients. The main research focus was set on the simplified model of a biphasic posttraumatic inflammatory response, with an initial systemic (pro)-inflammatory response syndrome (SIRS), and a “counterbalancing” compensatory anti-inflammatory response syndrome (CARS) [4, 6]. This theory has been widened to a simultaneous SIRS-CARS paradigm and for further ongoing injury-caused inflammatory processes. The central pathophysiological principle was set there on regulating the balance between a qualitative defense against invasive putative pathogens, and additionally reducing collateral damage by immune cells [10–12]. This biological host response to trauma including a massive cytokine release with activation and recruitment of effector cells of the immune system further employs a large number of both microbial pathogen-associated molecular patterns (PAMPs) and host alarmins danger-associated molecular patterns (DAMPs) [13–24]. Although a large number of endogenous nuclear or cytosolic triggers has been described in the context of the local/systemic posttraumatic and/or noninfectious inflammatory response, which represent key drivers of the late occurring post-injury complications and fatal outcome rates, the knowledge on their variety as well as their precise mechanisms still remains obscure [25–27]. Both in vitro and in vivo modeling of the complex injury patterns of polytrauma and the subsequent immune response remain difficult. Although valid and reliable murine and porcine animal models have been developed within the NTF group [28–30], the translational process to the heterogenous situation in the clinical setting of polytrauma remains crucial. Noteworthy, despite improved treatment strategies with regard to traumatic injuries as well as to the posttraumatic immune response, both mortality and disability rates remain alarmingly high.

To further decrypt the complexity of the posttraumatic immune responses and to potentially identify novel research pathways for exploration, large-scale multi-center projects including not only in vivo and in vitro modeling, but also temporal sample and material collection along with clinical data capture from multiple injured patients is of utmost importance. To meet this gap, a nationwide biobank for fluidic samples from polytrauma patients was initiated in 2013 by the NTF in Germany. The concept behind this idea is to create a robust interface for basic, translational and clinical research. This project was introduced to systemically evaluate and monitor multiple injured patients for their (patho-)physiological sequalae together with their clinical treatment strategies applied for overall outcome improval. Over the last 6 years the NTF-Biobank idea was significantly moved foreward. An ethical approval for the NTF-Biobank set-up and the sample collection was obtained, and funding for the pilot phase was secured by the German Trauma Society (DGU). The biological sampling process starts as early as possible at the admission of the patient to the emergency department and integrates a follow-up period of up to 10 days. The sampling of serum and plasma samples is performed immediately upon admission of the patient to the emergency department, after 8 h and at post-injury days 1, 2, 5, and 10. Inclusion criteria are: ≥ 18 years of age, injury severity score (ISS) ≥ 16, preclinical time < 120 min, regular finalization of the shock room phase, signed informed consent form. Exclusion criteria are: < 18 years of age, injury severity score (ISS) < 16, infaust prognosis within 24 h, cardiopulmonary reanimation at the trauma site, death immediately upon admission, gravidity, radio- or chemotherapy during the last 3 months, immunosuppressive medication, HIV, hepatitis A, B, C, HCM, CMV, kidney dialysis. The NTF-Biobank samples are stored centrally at the Department of Trauma, Hand and Reconstructive Surgery at the University Hospital of the Goethe University in Frankfurt. A back-up storage is planned at the Institute of Clinical- and Experimental Trauma-Immunology, University of Ulm. The sample storage as well as its inventory management is secured in close collaboration with the Interdisciplinary Biomaterial Bank and Database Frankfurt (iBDF), a member of the German Biobank Alliance. A long-term collaboration with the iBDF will support a pioneering collaborative effort towards the professionalization of the NTF-Biobank, its implementation in additional functional national networks and the development of IT infrastructures in an international context. The management and clinical annotation of biomaterials are organized centrally by the PI project management team using a powerful IT solution (CentraXX). With this software, biomaterial is annotated with necessary data, providing a valuable resource for clinicians and lab-based scientists for the development of new diagnostic and therapeutic approaches. A scientific board of the NTF allocates the biomaterials to scientific projects as described in the defined by-laws of the NTF.

The DGU also provided funding for the development of the corresponding online NTF-Biobank Database. The corresponding NTF-Biobank Database module is conceptualized as an add-on module to the well-established TraumaRegister DGU^®^ (TR-DGU) and technical translation and support was provided by the AUC—Academy for Trauma Surgery (AUC—Akademie der Unfallchirurgie GmbH). At present, the module is undergoing online testing within the TR-DGU framework, which is the representative registry for the care of severely injured patients in Germany [31, 32]. The AUC was founded in 2004 as a subsidiary of the DGU and constitutes as an innovative medical service company at the interface of clinical medicine, health research, quality assurance and management. The AUC is operating the TR-DGU, which is considered as one of the world’s largest medical trauma registries. Since 1993, the TR-DGU has documented the prehospital phase, treatment and outcome of 325.000 severely injured patients (status: end of 2017). At present, around 30,000–35,000 new cases are documented and added annually to the registry. In 2017, more than 650 hospitals entered data into the TR-DGU for both scientific and quality assurance purposes. The performance of each participating hospital is documented and benchmarked in an individual quality report [31]. The scientific analyses of the dataset resulted in nearly 300 peer-reviewed publications [33] and the findings found entry into guidelines and white papers for the treatment of severely injured patients [33, 34]. Derived measures and initiatives, for example, the close cooperation and systematic communication between hospitals providing different levels of care form the profound basis for currently 55 regional trauma networks which have led to reduction in both mortality and regional differences in outcome [32, 34, 35]. The data entry into the TR-DGU is mandatory for all certified trauma centers within TraumaNetzwerk DGU^®^ in a basic version, but offers the option for an extended dataset for additional scientific studies [34]. The NTF-Biobank together with its linked NTF-Database module will be part of the TR-DGU and will also be cross-linked to the newly implemented TBI-specific submodule to the TR-DGU to amalgamate clinical with analytical data (Fig. 1). The concept behind these specific add-on modules is to expand the clinical dataset of the TR-DGU which is used for quality control with spatial–temporal biological samples to generate an high-quality interface between basic, translational and clinical research with the aim of optimizing the treatment guidelines (Fig. 2) [36].

## Future

The NTF-Biobank completes the clinical NTF-Biobank Database and complements the TR-DGU with temporal biological samples from multiply injured patients. For the first time, an integrated platform to prospectively evaluate and monitor candidate biomarkers and/or potential therapeutic targets in biological specimens of quality-controlled and documented patients is introduced, allowing reduction in variability of measurements with high impact due to its large sample size. The feasibility of the multi-center NTF-Biobank as well as the online tool have been successfully proven and established in seven trauma centers during a pilot phase of 5 years which ended in 2018. There are two main goals to the multi-center NTF-Biobank. First, the further development, routine use and continuous inclusion of additional trauma centers along with the international expansion of the NTF-Biobank and –Database in addition to the ongoing polytrauma clinical data documentation and evaluation. Second, to offer all research partners and project applicants access to clinical and biological data for meaningful innovative translational research, and thereby to accelerate development and improvement of diagnostic methods and therapies for overall advance in medical progress. The NTF-Biobank has an established steering and review board and fulfills all ethical and data protection requirements that are currently relevant. This project represents a key component at the interface between basic, translational and clinical research and beyond the application.
